# A challenging case of transvenous pacemaker implant in a patient with anomalous pulmonary venous drainage

**DOI:** 10.1016/j.hrcr.2025.08.011

**Published:** 2025-08-16

**Authors:** Yi Lung Gan, Ahmed Mohamed, Howard Marshall, Francisco Leyva, Peysh Patel

**Affiliations:** 1 Department of Cardiology, Queen Elizabeth Hospital Birmingham, Birmingham, United Kingdom; 2 Aston Medical School, Aston University, Birmingham, United Kingdom

**Keywords:** Partial anomalous pulmonary venous drainage, Hypertrophic cardiomyopathy, Left bundle branch block, Cardiac resynchronization therapy, Heart failure

**Key Teaching Points**

• Partial anomalous pulmonary venous drainage is a congenital cardiac anomaly and is often a diagnosis of an incidental finding, as depicted in our case.
• Reviewing any previous imaging before the planned device procedure is crucial to exclude potential anatomical variants that may pose technical challenges and to facilitate preprocedural planning.
• During encounters with technical difficulties in pacemaker implantation, having a list of differentials and systematically working through them, such as looking at previous imaging, using contrast, or asking for a second opinion, can help prevent complications and improve procedural success.

## Introduction

Partial anomalous pulmonary venous drainage (PAPVD) is a congenital heart disease that can often remain undiagnosed because of its asymptomatic nature. The prevalence was thought to be around 0.4%–0.7% in the adult population.^1^ We report a case of right superior pulmonary venous drainage into the superior vena cava (SVC) in a patient with known hypertrophic cardiomyopathy and impaired left ventricular systolic function. The anomalous drainage pattern was identified during device implantation, necessitating real-time troubleshooting. Contrast fluoroscopy played a critical role in delineating the aberrant anatomy, guiding wire manipulation through the SVC while avoiding entry into the right pulmonary vein.

## Case report

A 62-year-old gentleman with known diabetes mellitus, atrial fibrillation, hypertension, and previous myocardial infarction was diagnosed with hypertrophic cardiomyopathy in 2012, as the echocardiogram showed chordal systolic anterior motion and cardiac magnetic resonance confirmed the diagnosis. His gene panel was negative for any mutation. Unfortunately, he was lost to follow-up since then and presented to a different hospital with worsening heart failure symptoms, including shortness of breath with both lower limbs swelling since November 2024. His heart failure medication included bisoprolol, dapagliflozin, sacubitril/valsartan, and spironolactone, which were already optimized.

**Figure 1:**
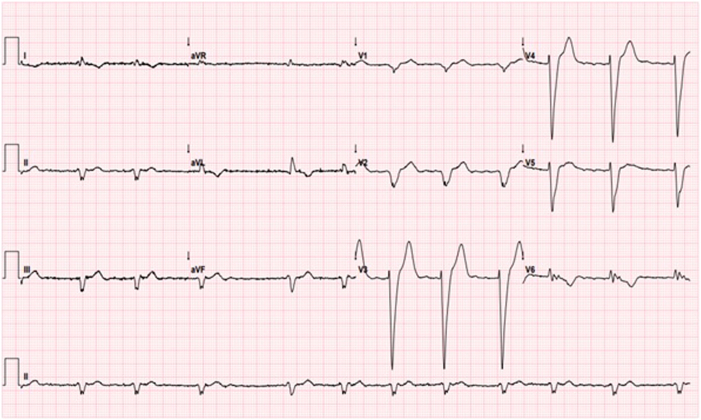
Twelve-lead electrocardiogram showing rate-controlled atrial fibrillation with broad left bundle branch block.

**Figure 2:**
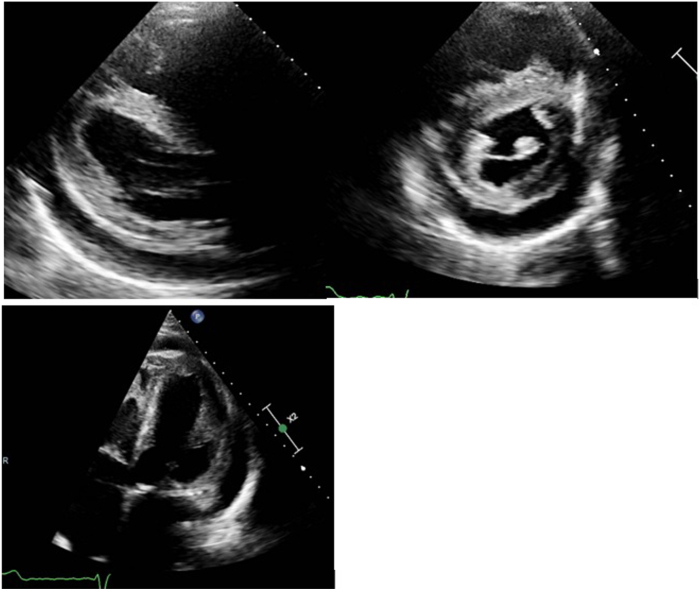
Transthoracic echocardiogram showing left ventricular hypertrophy with global pericardial effusion.

**Figure 3:**
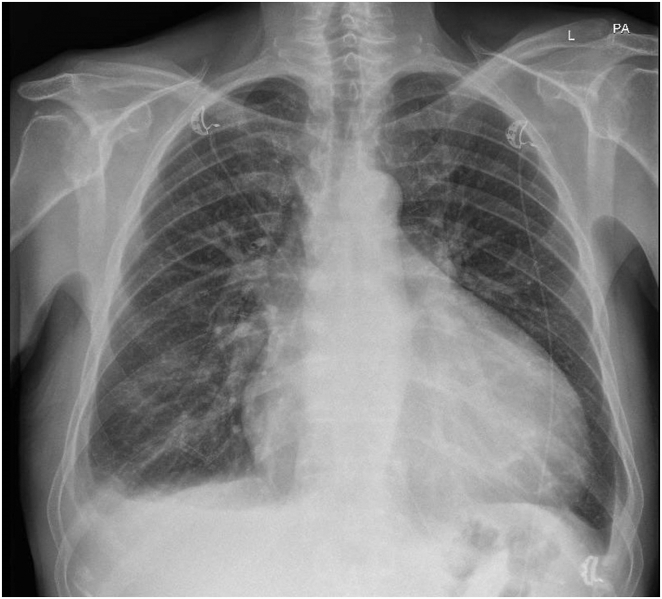
Chest radiograph showing cardiomegaly and right-sided pleural effusion.

He was transferred back to our care in February 2025, as he was readmitted with heart failure decompensation. Examination revealed signs suggestive of congestive cardiac failure, including an elevated jugular venous pressure of 6 cm and bilateral lower limbs edema up to the knee level. Chest examination showed no crepitation or murmur. The electrocardiogram showed known atrial fibrillation with a rate of 67 beats/min and broad left bundle branch block morphology with a QRS duration of 176 ms (Figure 1). The echocardiogram showed concentric left ventricular hypertrophy with no significant outflow tract obstruction with a pressure gradient of 19 mm Hg. The left ventricular ejection fraction was 35%. The right ventricle was dilated, with an indexed end-diastolic area of 17 cm^2^/m^2^, impaired systolic function, and a high probability of pulmonary hypertension. There was biatrial dilatation. There was also moderate global pericardial effusion, largest at the anterolateral side of the left ventricular free wall measuring 1.5 cm, with no evidence of hemodynamic compromise (Figure 2). There was no evidence of any obvious left-to-right shunt, such as atrial septal defects or sinus venosus defects. A chest radiograph showed cardiomegaly and right-sided pleural effusion (Figure 3).

Given his refractory heart failure symptoms despite optimal medical therapy, implantation of a cardiac resynchronization therapy device via a transvenous route was planned. This decision was made in line with local guidelines after a multidisciplinary team discussion. It was based on the presence of broad left bundle branch block morphology on the electrocardiogram, which suggested that he would benefit from improved ventricular synchrony through cardiac resynchronization therapy.

**Figure 4:**
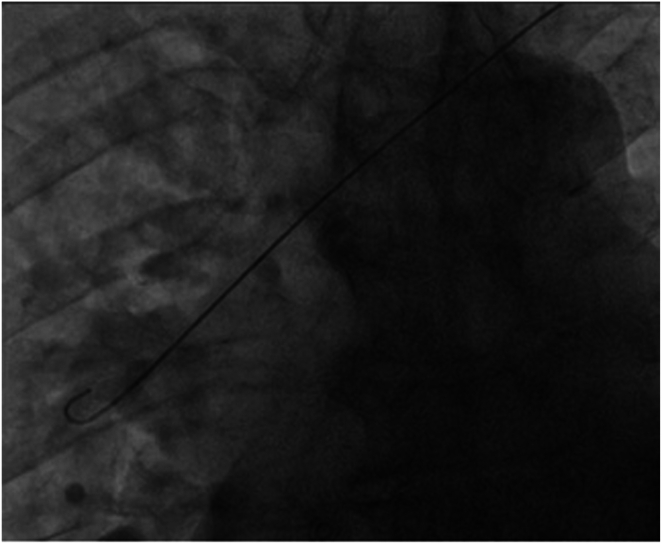
Fluoroscopy showing guidewire crossing the superior vena cava into the right-sided vein bypassing the right atrium.

**Figure 5:**
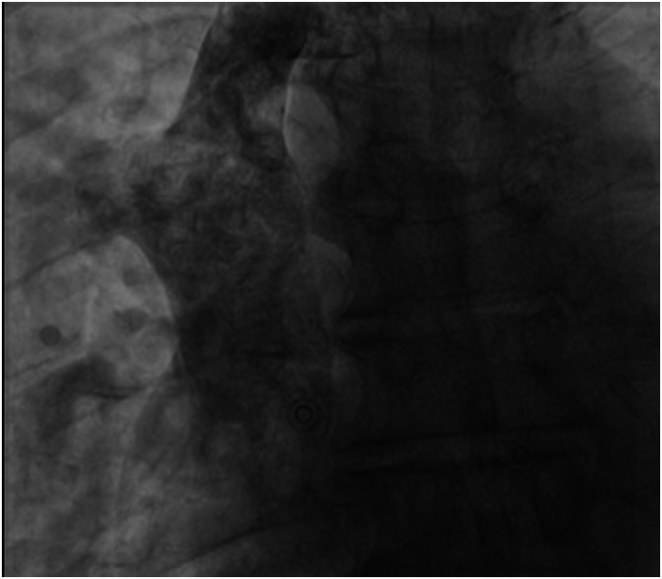
Fluoroscopy demonstrates the presence of a right-sided vein draining into the superior vena cava.

The procedure was undertaken under conscious sedation. An incision was made at the left infraclavicular region, and vascular access was obtained via the left axillary vein under fluoroscopy-guided venography. The initial passing of the guidewire did not take the usual route of SVC into the right atrium (RA), and instead, the guidewire entered the SVC and then into a right-sided vessel outside the cardiac shadow, as shown by fluoroscopy, suggesting that the course was extracardiac (Figure 4). As the route was unconventional, we proceeded with contrast injection further down the level of SVC, which showed a large right-sided vessel draining into the SVC that did not represent the usual anatomical position of the right brachiocephalic vein, as the level was too low. Furthermore, this vein drained from the inferior part of the right lung, as suggested by the position of the guidewire (Figure 5).

**Figure 6:**
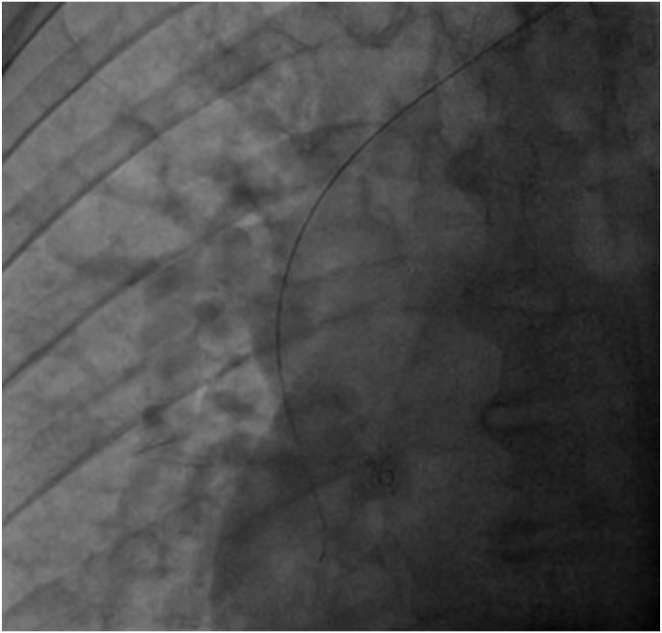
Fluoroscopy demonstrates the maneuver of the guidewire into the right atrium.

**Figure 7:**
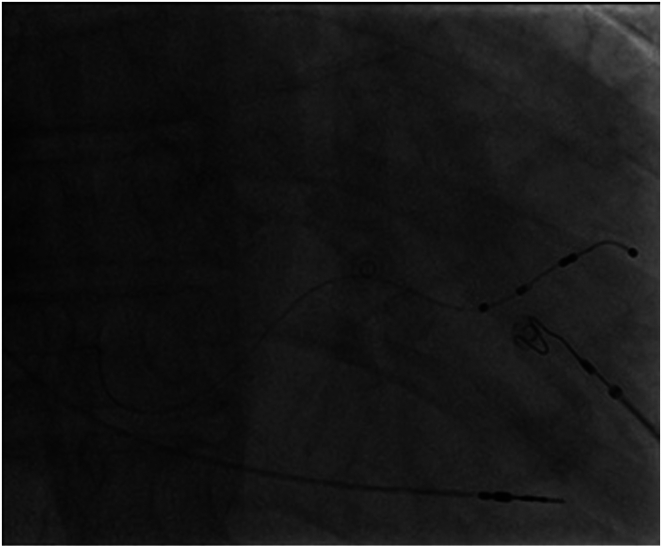
The result of the procedure showing successful placement of a right ventricular lead in the right ventricular (RV) apex and a left ventricular lead in the posterolateral vein.

We then proceeded to use a long guidewire with a 23-cm long sheath through the left axillary access to pass the unusual venous drainage–SVC junction into the SVC, which then enabled us to wire into the true lumen of the SVC-RA junction (Figure 6). The procedure was performed without difficulties once wire access into the RA has been established. The procedure was successful, albeit with technical challenges in guidewire manipulation owing to abnormal venous runoff. We were able to secure the right ventricular lead in the right ventricular apex and the left ventricular lead in the posterolateral vein with good pacing parameters. The result is shown in Figure 7.

**Figure 8:**
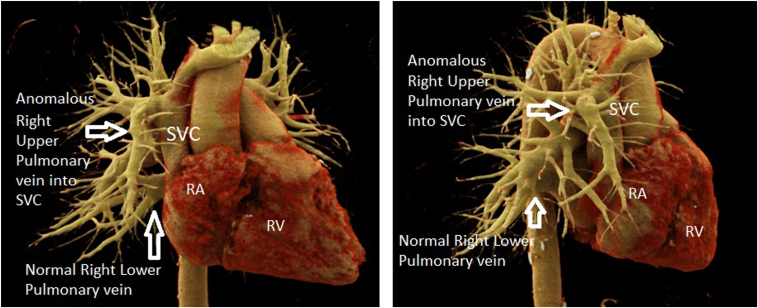
Three-dimensional computed tomography reconstruction showing an anomalous right upper pulmonary vein draining into the superior vena cava (SVC). RA = right atrium; RV = right ventricle.

After the procedure, 3-dimensional computed tomography (CT) reconstruction confirmed that the right superior pulmonary vein drained into the SVC. The right inferior and left pulmonary veins drained normally into the left atrium. There were no other cardiac anomalies (Figure 8). This confirmed the diagnosis of PAPVD of the right superior pulmonary vein into the SVC.

## Discussion

PAPVD is a congenital cardiac condition whereby 1 or more pulmonary veins return to the RA or any systemic venous drainage instead of the left atrium. It is a form of a left-to-right shunt that may lead to right ventricular dysfunction and pulmonary hypertension.^2^ It is rare, accounting for less than 1% of all congenital heart diseases.^3^ Most patients remain asymptomatic and thus, remained undiagnosed until further imaging modalities are performed for other indications, resulting in its incidental finding.

In our patient, we demonstrated a case of PAPVD of the right upper pulmonary vein into the SVC without other congenital cardiac anomalies. In the literature and case reports, partial anomalous venous drainage into the SVC is often associated with a sinus venosus defect.^4^ This was absent in our case, as no other septal defects were found on imaging.

CT or magnetic resonance imaging remains the mainstay imaging modality to diagnose PAPVD. It allows visualization of the pulmonary venous drainage anatomy and other associated abnormalities. The 3D CT reconstruction as shown in our case, during venous phase contrast injection, provided a clear anatomical delineation of the PAPVD of the right pulmonary vein into the SVC and confirmed that no other significant anomalies were present (Figure 8).

During pacing, the presence of anomalous drainage into the SVC may pose challenges to the operators, as demonstrated in this case where the guidewire may not directly enter the RA. This raises the suspicion about whether the access site puncture is arterial or venous or whether there is abnormal venous drainage. This highlights the importance of having a systematic approach by stopping and thinking through the differentials. Exchanging to a smaller and more flexible wire may allow easier wire manipulation. The use of contrast fluoroscopy in the presence of an unusual wire position may demonstrate the anatomy of the venous drainage, such as partial anomalous venous drainage into the SVC or a persistent left SVC. The use of contrast also allows us to determine whether the access is arterial or venous, as contrast injection through an arterial access will show an aortogram. It is imperative to avoid sheath insertion until we are certain that the access site is not arterial to prevent bleeding complications. When in doubt, reviewing previous imaging, such as CT, may be helpful if this has been done in the past. Once the wire has successfully entered the RA, using a long sheath to secure access to the RA makes the remaining procedure easier.

## Conclusion

The right pulmonary vein draining into the SVC is a form of PAPVD. When faced with challenging and uncertain anatomy during device implantation, a systematic approach—walking through the differentials and reviewing previous imaging—can be helpful. The use of a longer and smaller guidewire may allow easier manipulation. Contrast injection during the procedure provides invaluable information, confirming arterial or venous access or abnormal venous drainage.

## Disclosures

All authors have no conflicts to disclose.
